# Total Knee Arthroplasty With and Without Schedule II Opioids: A Randomized, Double-Blinded, Placebo-Controlled Trial

**DOI:** 10.7759/cureus.56150

**Published:** 2024-03-14

**Authors:** Porter F Young, Christopher Roberts, Glenn G Shi, Michael G Heckman, Launia White, Steven Clendenen, Benjamin Wilke

**Affiliations:** 1 Orthopedic Surgery, University of Florida, Jacksonville, USA; 2 Orthopedic Surgery, Mayo Clinic, Jacksonville, USA; 3 Orthopedics, Mayo Clinic, Jacksonville, USA; 4 Biostatistics, Mayo Clinic, Jacksonville, USA; 5 Anesthesiology and Perioperative Medicine, Mayo Clinic, Jacksonville, USA

**Keywords:** postoperative pain control, opioid, pain after total knee arthroplasty, multimodal pain control, total knee arthroplasty (tka)

## Abstract

Introduction:Orthopedic surgeons are the third highest prescribers of narcotics. Previous work demonstrated that surgeons prescribe three times the narcotics required, and most patients do not properly dispose of leftover medication following surgery. This has prompted the creation of multimodal pain regimens to reduce reliance on narcotics. It is unknown if these pathways can effectively eliminate opioids following total knee arthroplasty (TKA). Our purpose was to evaluate a multimodal regimen without schedule II narcotics following TKA, in a randomized, blinded fashion. We hypothesized that there would be no difference in pain scores between groups.

Methods:A total of 43 narcotic-naïve patients participated in a randomized, double-blinded, placebo-controlled trial. Postoperative protocols were identical between cohorts, except for the study medication. The narcotic group received an encapsulated 5 mg oxycodone, whereas the control group received an encapsulated placebo. Perioperative outcomes were compared with routine statistical analysis.

Results: Four patients withdrew early secondary to pain: three in the placebo group and one in the narcotic group (p=1.00). We found no difference in hospital length of stay (p=0.09) or pain scores at all time points between cohorts (all p>0.05). There was a higher proportion of patients using a narcotic in the opioid treatment arm at day 30 (40% vs. 21.4%, p=0.29) and day 60 (20% vs. 7.1%, p=0.32), although this was not statistically significant.

Conclusion: A multimodal regimen without schedule II narcotics demonstrates equivalent pain scores and may reduce the risk of long-term opioid dependence following TKA.

## Introduction

The United States continues to experience a rapid increase in opioid abuse. In 2017 alone, the Centers for Disease Control and Prevention (CDC) estimated that 72,000 deaths in the United States were related to drug overdose, with many of these stemming from prescribed opioids. This was an approximate twofold increase in mortality compared to the previous decade and did not include the additional two million who suffer from a substance abuse disorder related to opioid medications [1]. Further, the National Survey on Drug Use and Health estimated that more than 10 million people in the United States used opioids outside of their prescribed intent, with 55% of the medication obtained from a friend or relative [2]. These alarming statistics have forced both state and federal governments to declare an opioid epidemic and enact laws to limit opioid prescriptions [3].

The high rate of opioid over-prescription following surgery is thought to contribute to the current epidemic [4]. Orthopedic surgeons are the third highest opioid prescribers [4-7]. Previous work demonstrated that orthopedic surgeons prescribe three times the amount of opioids required by the patient and that most patients do not properly dispose of leftover medication [8-11]. Additionally, in separate studies, 11-14% of opioid-naïve patients became prolonged users following elective orthopedic procedures [12,13]. Therefore, there is a critical need to find alternative approaches to pain management that rely less on opioid medications following surgery to mitigate the amount of opioids available for diversion.

Multimodal pain regimens have gained popularity in recent years in an effort to improve postoperative pain control while simultaneously reducing reliance on opioid medications [14-18]. These regimens utilize local anesthetics, peripheral nerve blocks, and non-narcotic alternatives for effective pain control. Multimodal regimens have been shown to improve postoperative pain while also limiting the negative side effects of opioids [10,11,17,18]. It is currently unknown, however, if these multimodal regimens can effectively control postoperative pain without the inclusion of schedule II opioids. The purpose of our randomized controlled trial was to evaluate a multimodal pain regimen following total knee arthroplasty (TKA) with and without schedule II opioids (oxycodone) via a blinded, placebo-controlled approach. Our aims included the following: (1) compare the average postoperative pain levels both in the hospital setting and daily for two weeks following discharge, between patients in the opioid treatment group and those in the placebo group; (2) compare the oral morphine equivalents (OMEs) used during hospitalization between the opioid and placebo treatment groups; (3) compare postoperative constipation, nausea, and vomiting between treatment groups; and (4) compare the length of post-surgery hospitalization between treatment groups.

Our hypotheses were that the placebo group would demonstrate equivalent pain control compared to the opioid group with lower rates of postoperative constipation, nausea, and vomiting and equivalent length of hospitalizations.

This article was previously presented at the 2021 American Academy of Orthopaedic Surgeons (AAOS) Annual Meeting.

## Materials and methods

After institutional review board (IRB) approval from the Mayo Clinic IRB (approval number: 18-004256), the study was registered on ClinicalTrials.gov on February 19, 2019 (NCT03845881) and followed the Consolidated Standards of Reporting Trials (CONSORT) guidelines. Inclusion criteria included the following: (1) >18 and <90 years of age, (2) willing to participate in the study and competent to provide informed consent, (3) willing to comply with protocol procedures, and (4) had an underlying diagnosis of osteoarthritis and was indicated for a TKA.

Patients were excluded for the following: (1) had a diagnosis of renal or liver disease, (2) had a contraindication to receiving a spinal anesthetic or pain catheter, (3) had taken any schedule II-IV opioid medications within three months prior to enrollment, (4) had an allergy or intolerance to a medication used in the multimodal pain pathway, (5) was undergoing a revision procedure, (6) was being treated under worker's compensation, (7) had a diagnosis of diabetes, (8) was unable to take aspirin 81 mg, twice daily for deep venous thromboprophylaxis, and (9) planned to discharge to a skilled nursing facility. 

Our aims included the following: (1) compare the average postoperative pain levels both in the hospital setting and daily for two weeks following discharge, between patients in the opioid treatment group and those in the placebo group, (2) compare the OMEs used during hospitalization between the opioid and placebo treatment groups, (3) compare postoperative constipation, nausea, and vomiting between treatment groups, and (4) compare the length of post-surgery hospitalization between treatment groups.

Eligible patients were screened and consented to the Mayo Clinic Florida campus, beginning April 10, 2019, until March 2020. Opioid history was checked through the state prescription drug monitoring database to verify eligibility. Once enrolled, patients were randomized to either the opioid (oxycodone) or placebo group using computer-based randomization stratified by gender and pre-operative Numerical Rating Scale (NRS scores 1-5 and 6-10). In May 2019, after enrollment commenced, a revision to the protocol was made to allow diabetic patients, who were initially excluded due to a concern for elevated perioperative blood glucose levels, to participate in the study if their hemoglobin A1c values were less than 8%.

**Figure 1:**
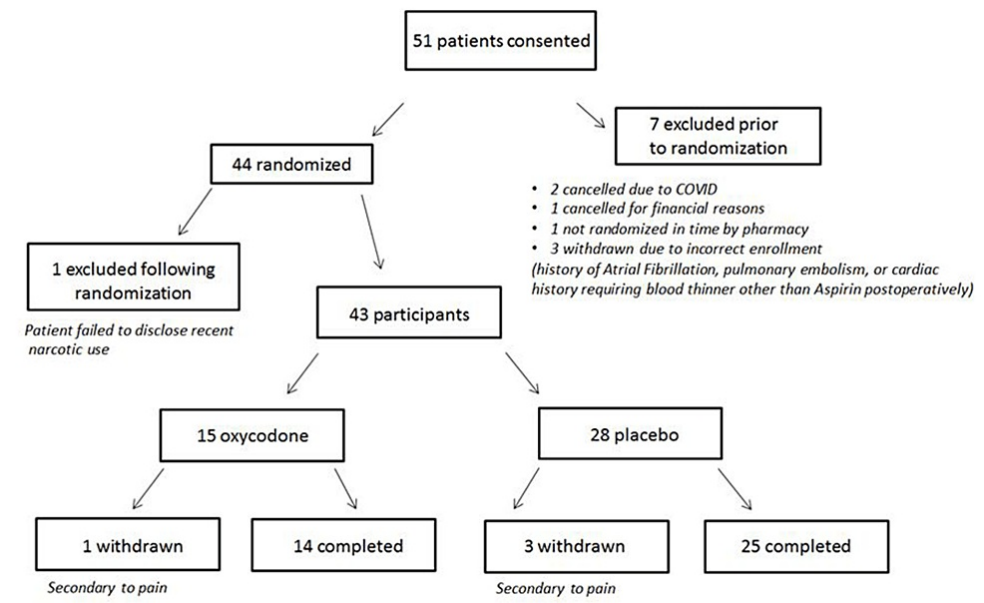
Flowchart of patients consented and included in the trial

Our study had planned for a total of 50 patients to be enrolled in the study, in a 1:1 fashion. Due to the preliminary nature of the study, this sample size was chosen based on logistical and feasibility issues rather than on formal statistical power calculations. Due to the onset of coronavirus disease 2019 (COVID-19) and the cessation of research activities at our institution, enrollment was stopped early. At the time of stoppage, 51 patients had consented to the study. Seven patients were excluded prior to randomization, and one additional patient was excluded following randomization, yielding a final sample size of 43 patients (15 opioid, 28 placebo) (Figure 1). The imbalance in sample size between the two treatment groups was caused by the combination of the aforementioned early stop to enrollment and the stratified randomization.

All procedures were performed by a single surgeon (BKW), and there were no differences in surgical technique between groups. All patients underwent a medial parapatellar approach with patellar resurfacing. Each patient received an intraoperative periarticular injection consisting of 30 mg of ketorolac along with weight-based ropivacaine and epinephrine. The medication was diluted in normal saline to a final volume of 120 milliliters and injected into the soft tissues surrounding the knee prior to wound closure, including the posterior capsule, femoral periosteum, and subcutaneous tissue. An adductor catheter was placed by the anesthesia team postoperatively in the recovery room under ultrasound guidance with an initial bolus of 10 mL of 0.5% ropivacaine. The catheter was infused with ropivacaine 0.2% at a rate of 6 mL/hr, with an additional hourly patient-controlled on-demand bolus of 6 mL (Ambit; Summit Medical Products, Sandy, Utah, United States). Patients were discharged with the catheter in place, and daily phone calls were made to the patient by the acute pain team. The catheters were removed by the patient or family member on postoperative day (POD) 4 with no reported complications.

The postoperative multimodal regimens are listed in the Appendices. The treatment arms were identical, with the exception of the study medication; this was either a placebo pill or a 5 mg oxycodone pill. The study medication was encapsulated by the research pharmacy prior to dispensing; thus, all participants in the study were completely blinded, including the nursing staff, surgical team, and patient. Placebo capsules were filled with microcrystalline cellulose: 0.2 grams per capsule. The oxycodone capsules each contained one 5 mg oxycodone tablet plus 0.15 grams of microcrystalline cellulose. Capsules were size 0 orange gelatin capsules.

At the time of discharge, the patient was provided with a two-week prescription of their encapsulated study medication (50 pills). The research team collected daily questionnaires for POD 1-14, followed by repeat questionnaires at 30 and 60 days postoperatively. These questionnaires recorded average daily NRS pain scores and symptoms of nausea, vomiting, and constipation. If a patient failed to fill out the questionnaire within four hours, the research team was notified, and a member of the team contacted the patient to collect the data.

The primary outcome in the study was pain calculated using NRS scores. Secondary outcomes included OMEs consumed during the hospitalization, hospital length of stay (LOS), incidence of postoperative nausea, vomiting, and constipation, and duration of study medication use (or alternate opioid for patients that withdrew). OMEs included all opioid medication consumed during the postoperative hospitalization, including IV medications. Patients were withdrawn from the study and provided an opioid prescription if they reported pain that they felt was uncontrolled with the multimodal regimen. Patients were also withdrawn if they required additional opioid refills prior to the study's conclusion. Additionally, at the conclusion of the study, patients were asked to identify in which treatment group they were believed to have been assigned.

Statistical analyses were performed based on the intention-to-treat principle. Continuous variables were summarized with the sample mean and range. Categorical variables were summarized with the number and percentage of patients. Comparisons of baseline characteristics and outcomes between the placebo and opioid groups were made using a Wilcoxon rank-sum test (continuous and ordinal variables) and Fisher's exact test (categorical variables). After excluding patients who stated that they did not take the trial medication, the degree of agreement between actual treatment assignment and patient hypothesis regarding treatment assignment was evaluated by estimating the proportion of agreement and also by estimating the kappa statistic. No adjustment for multiple testing was made due to the preliminary nature of this study; therefore, p-values <0.05 were considered statistically significant. All statistical tests were two-sided, and analyses were performed using SAS (Version 9.4; SAS Institute, Inc., Cary, North Carolina, United States).

## Results

A comparison of baseline patient characteristics between the placebo and opioid arms is shown in Table 1. Previous ipsilateral knee surgery was less common for the opioid group compared to the placebo group (n=4, 27% vs. n=18, 64%, p=0.027). There were no other significant differences in baseline characteristics between the two treatment groups.

**Table 1:** Baseline characteristics for the oxycodone and placebo treatment groups The sample mean (minimum, maximum, or percentage) is given for continuous variables. P-values result from a Wilcoxon rank-sum test (continuous variables) or Fisher's exact test (categorical variables). Information was unavailable regarding race (N=1). P<0.05 is considered significant. BMI: body mass index; NSAIDs: nonsteroidal anti-inflammatory drugs; NRS: Numerical Rating Scale

Variable	Oxycodone (N=15)	Placebo (N=28)	P-value
Age at surgery (years)	69 (60, 80)	64 (35, 78)	0.14
Sex (male)	6 (40%)	15 (53.6%)	0.53
Race (White)	15 (100%)	25 (92.6%)	1.00
BMI	30.7 (22.6, 38.9)	30.5 (15.5, 49)	0.73
Previous surgery	4 (26.7%)	18 (64.3%)	0.027
Currently taking NSAIDs	8 (53.3%)	20 (71.4%)	0.32
Previous knee injections	13 (86.7%)	23 (82.1%)	1.00
NRS pain score	5.6 (3, 9)	4.9 (2, 10)	0.32
Laterality (right)	7 (46.7%)	20 (71.4%)	0.19

**Figure 2:**
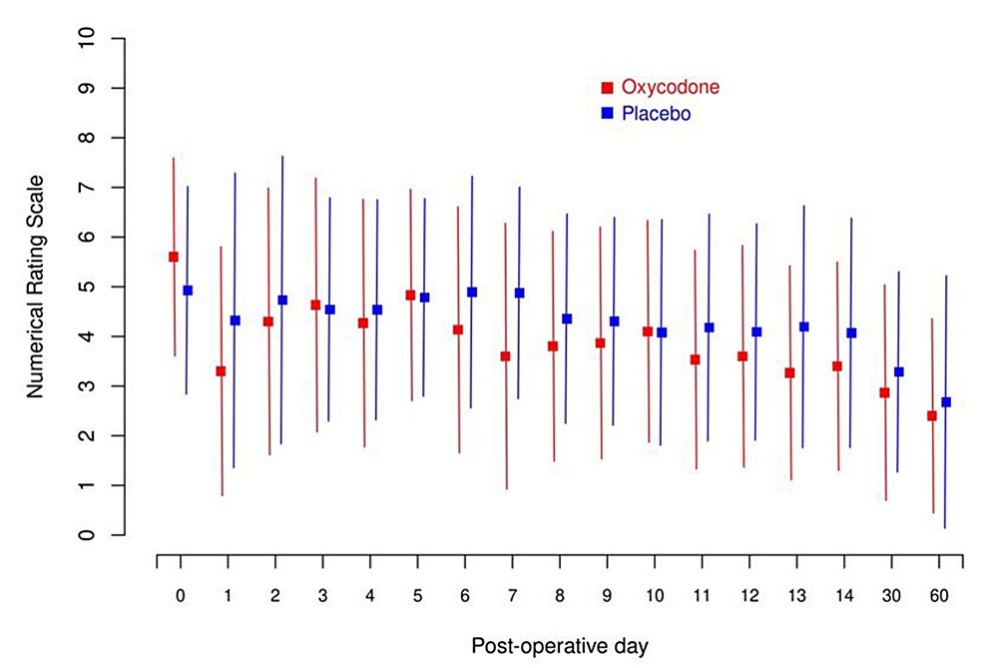
Mean NRS pain score on each postoperative day for the oxycodone and placebo treatment groups. Error bars represent the standard deviation of the NRS pain score on the given postoperative day for the given treatment group NRS: Numerical Rating Scale

Outcomes are compared between the two treatment groups in Table 2. We found no major differences between the opioid and placebo groups regarding OMEs received during hospitalization (mean: 31 vs. 23, p=0.70), use of in-hospital IV pain medication (n=2, 13.3% vs. n=7, 25%, p=0.458), incidence of nausea, constipation, or vomiting, or hospital LOS (mean: 1.1 vs. 0.9 days, p=0.095). NRS pain scores did not differ significantly between the opioid and placebo groups when considering the average pain score from POD 1 to 14 (mean: 3.9 vs. 4.5, p=0.42), POD 14 (mean: 3.5 vs. 4.1, p=0.40), POD 30 (mean: 2.9 vs. 3.4, p=0.53), or POD 60 (mean: 2.5 vs. 2.8, p=0.74). However, when examining changes in NRS scores compared to the baseline score, there was a greater decrease in the opioid group, significant only on POD 14 (mean: -2.1 vs. -0.8, p=0.037) and POD 30 (mean: -2.7 vs. -1.5, p=0.042). A summary of the NRS pain scores is shown in Figure 2.

**Table 2:** Comparison of outcomes between the oxycodone and placebo treatment groups The sample mean (minimum, maximum, or percentage) is given for continuous variables. P-values result from a Wilcoxon rank-sum test (continuous and ordinal variables) or Fisher's exact test (categorical variables). "Group patient believes they were in" was the group that the patient believed they were randomized to, based on a postoperative survey. Information was unavailable regarding the group the patient believed they were in (N=1), nausea that interfered with activities of daily living (N=1 post-discharge days 1-14, N=2 post-discharge days 1-60), and still using study pain medication (N=1 on both post-discharge days 30 and 60). For the four patients who withdrew, the "still using study pain medication" variable represents whether they were still using narcotics. P<0.05 is considered significant. OMEs: oral morphine equivalents; NRS: Numerical Rating Scale

Variable	Oxycodone (N=15)	Placebo (N=28)	P-value	
Withdrawn	1 (6.7%)	3 (10.7%)	1.00
OMEs received during hospitalization	31 (0, 105)	23 (0, 55)	0.70
Length of hospital stay (days)	1.1 (1, 2)	0.9 (0, 2)	0.095
Group patient believes they were in			0.51
Oxycodone	4 (28.6%)	6 (21.4%)	-
Placebo	6 (42.9%)	17 (60.7%)	-
Never took trial medications	4 (28.6%)	5 (17.9%)	-
NRS pain score			
Average during post-discharge days 1-14	3.9 (0.4, 7.5)	4.5 (0.4, 10)	0.42
Post-discharge day 14	3.5 (0, 8)	4.1 (0, 10)	0.40
Post-discharge day 30	2.9 (0, 8)	3.4 (0, 10)	0.53
Post-discharge day 60	2.5 (0, 8)	2.8 (0, 10)	0.74
NRS pain score: change from baseline			
Average during post-discharge days 1-14 minus baseline	-1.7 (-4.6, 1.5)	-0.4 (-6.2, 3.4)	0.059
Post-discharge day 14 minus baseline	-2.1 (-5, 2)	-0.8 (-7, 3)	0.037
Post-discharge day 30 minus baseline	-2.7 (-6, 2)	-1.5 (-8, 3)	0.042
Post-discharge day 60 minus baseline	-3.1 (-6, 2)	-2.1 (-10, 3)	0.080
Pain			
Any during post-discharge days 1-14	15 (100%)	28 (100%)	1.00
Post-discharge day 14	14 (93.3%)	24 (88.9%)	1.00
Post-discharge day 30	12 (80%)	26 (96.3%)	0.12
Post-discharge day 60	12 (80%)	20 (74.1%)	1.00
Constipation			
Any during post-discharge days 1-14	9 (60%)	19 (67.9%)	0.74
Any during post-discharge days 1-60	9 (60%)	19 (67.9%)	0.74
Vomiting/dry-retching			
Any during post-discharge days 1-14	3 (20%)	3 (10.7%)	0.65
Any during post-discharge days 1-60	4 (26.7%)	3 (10.7%)	0.22
Nausea			
Any during post-discharge days 1-14	4 (26.7%)	11 (39.3%)	0.51
Any during post-discharge days 1-60	5 (33.3%)	13 (46.4%)	0.52
Nausea that interferes with activities of daily living			
Any during post-discharge days 1-14	1 (6.7%)	5 (17.9%)	0.40
Any during post-discharge days 1-60	1 (6.7%)	6 (21.4%)	0.39
Still using study pain medication			
Post-discharge day 30	6 (40%)	6 (21.4%)	0.29
Post-discharge day 60	3 (20%)	2 (7.1%)	0.32

A total of four patients withdrew from the study, all due to pain issues: one patient (6.7%) was from the opioid treatment group, and three (10.7%) were from the placebo group (p=1.00). There was a higher proportion of patients who were still using the blinded study medication or alternate opioid pain medication (for those who withdrew) in the opioid group compared to the placebo group at POD 30 (n=6, 40% vs. n=6, 21.4%, p=0.29) and POD 60 (n=3, 20% vs. n=2, 7.1%, p=0.32), but this difference was not statistically significant.

Out of 43 patients, 10 (23%) reported never taking the study medication. After excluding those patients, an additional 10 (23%) patients believed they were assigned to the opioid treatment arm, while 23 (54%) patients felt they were assigned to the placebo arm. Overall agreement between actual treatment assignment and patient hypothesis regarding treatment assignment was poor to fair (kappa=0.14, 63.6% agreement); six of 10 patients (60%) in the opioid treatment group thought that they received a placebo medication, while six of 23 patients (26.1%) in the placebo group believed that they were utilizing opioid medication.

## Discussion

Opioid consumption has increased dramatically in the United States, resulting in the declaration of an epidemic and governing efforts to limit opioid over-prescribing [3,4]. With an expected continued increase in annual procedures, orthopedic surgeons have both the opportunity and obligation to help combat the opioid crisis [19]. In this randomized, placebo-controlled trial, we evaluated a multimodal regimen after TKA and were unable to find a statistically significant difference in pain scores or hospital LOS when schedule II opioids were eliminated from the postoperative pain management protocol.

In an effort to reduce opioid medications, surgeons have begun to rely on multimodal regimens consisting of non-opioid alternatives and peripheral nerve blocks. Several studies have reported improvements in pain control with these regimens [15,16,20-24]. In addition to oral medications, selected nerve blocks have been evaluated with varying results [25-27]. The authors previously evaluated our own multimodal regimen with and without an adductor canal catheter and reported a 43% decrease in in-hospital opioid use when the adductor catheter was used in combination with a local periarticular injection compared to a periarticular injection alone [14]. While these studies are important initial steps, they fall short of determining if the multimodal regimens are able to completely eliminate opioids, as was performed in the present study. In fact, there were only two time points (POD 14 and 30) where the opioid group demonstrated a significantly greater decrease in reported pain scores compared to the placebo group. However, as the minimum clinically important difference has been reported to be 1.3 for the NRS pain scale, which was not observed between the groups, this difference is clinically meaningless [28,29].

Shah et al. reported that increased initial prescriptions of opioid medications were associated with chronic use in patients [30]. In one study, 13% of opioid-naïve patients became chronic opioid users following elective orthopedic procedures [13]. Our findings were similar, with 20% of patients in the opioid group reporting use at 60 days postoperatively. This finding is concerning, especially considering that these patients reported similar pain scores to those who were not taking the opioid medication. Both the continued refinement of non-opioid medication regimens by prescribers and state laws limiting the duration of opioid prescriptions may help reduce excess opioid medication available for diversion and reduce the risk of long-term dependence [31-33].

At the conclusion of the study, participants were asked to predict in which group they believed they were randomized to. Ten patients (23%) never took the study medication and were unable to answer the question. Out of those who answered, 10 (23%) believed they were in the opioid group and 23 (54%) in the placebo group. Overall, the ability of patients to accurately determine if they were taking a schedule II opioid was poor. For this reason, a better understanding of non-opioid alternatives and intentional clinical follow-up of both the quantity and duration of any prescribed opioid by the surgical team is critical.

The main limitation of this study was the small sample size, which introduces the possibility of a type II error (false-negative finding); thus, it cannot be concluded that a difference does not exist in outcomes simply due to a non-significant p-value. Additionally, patients were only included if they were opioid-naïve, and the findings cannot be extrapolated to opioid-tolerant patients. Furthermore, tramadol use was not accounted for postoperatively. It is possible that patients consumed more tramadol as they reduced their oxycodone use. We view opioid reduction as an iterative process; reducing oxycodone use is simply the first step. Future studies will attempt to include tramadol in the reduction efforts. Finally, there was a disproportionate distribution between study arms. The imbalance in treatment groups was caused by the early discontinuation of the study due to the COVID-19 pandemic as well as our stratified design. Although this imbalance does result in lower power, it does not affect the validity of the randomized design.

## Conclusions

The results of this study suggest that a multimodal regimen without schedule II opioids demonstrates equivalent pain scores, hospital LOS, and incidence of nausea, constipation, and vomiting and may reduce the risk of long-term opioid dependence following TKA. Arthroplasty surgeons should continue to consider decreasing quantity or possibly eliminating opioid use after TKA. We hope that the results of this study can form the basis for larger trials applied more generally across a variety of orthopedic procedures.

## Appendices

List of medications in the multimodal regimen

All patients received the following medications preoperatively, in the holding bay: (1) acetaminophen 1,000 mg tablet orally, (2) Celebrex 400 mg orally, and (3) dexamethasone 10 mg intravenously. During the intraoperative phase of care, all patients received a spinal anesthetic and periarticular block. The periarticular block was weight-based and included the following: ketorolac 30 mg with weight-based ropivacaine and epinephrine, diluted in saline to a total volume of 120 mL.

Postoperatively, patients underwent adductor canal catheter placement in the recovery room. The catheter was bolused with 10 cc of 0.5% ropivacaine during placement. The pump then ran a 6 cc/hour infusion of 0.2% ropivacaine with an optional 4 cc/hour bolus as needed, for pain. The catheter remained in place for four days postoperatively. 

In addition to the catheter, patients received scheduled acetaminophen 1,000 mg orally every six hours, dexamethasone 10 mg intravenously daily (while hospitalized), and Toradol 10 mg orally every six hours (×3 days, while hospitalized). Patients also had the option of tramadol 50 mg orally every six hours as needed for pain, in addition to the encapsulated, blinded study drug (oxycodone 5 mg or placebo) every three hours as needed for pain. Patients had the option to receive 0.5 mg of IV Dilaudid every three hours as needed for pain >8 on a Visual Analog Scale. 

At discharge, patients were prescribed acetaminophen 1,000 mg orally every six hours scheduled and Celebrex 400 mg daily (both for two weeks). They also received tramadol 50 mg tablets, which could be taken every six hours as needed for pain, as well as the encapsulated, blinded study drug (oxycodone 5 mg or placebo), to be taken every three hours as needed for pain.

## References

[1] Rudd RA, Seth P, David F, Scholl L (2016). Increases in drug and opioid-involved overdose deaths - United States, 2010-2015. MMWR Morb Mortal Wkly Rep.

[2] Warner M, Chen LH, Makuc DM (2009). Increase in fatal poisonings involving opioid analgesics in the United States, 1999-2006. NCHS Data Brief.

[3] (2020). Injury prevention legislation database. https://www.ncsl.org/health/injury-prevention-legislation-database.

[4] Habermann EB (2018). Are opioids overprescribed following elective surgery?. Adv Surg.

[5] Volkow ND, McLellan TA, Cotto JH, Karithanom M, Weiss SR (2011). Characteristics of opioid prescriptions in 2009. JAMA.

[6] Labrum JT 4th, Ilyas AM (2017). The opioid epidemic: postoperative pain management strategies in orthopaedics. JBJS Rev.

[7] (2020). Combatting opioid misuse. https://www.aaos.org/AAOSNow/2017/Jun/YourAAOS/youraaos10/.

[8] Scully RE, Schoenfeld AJ, Jiang W (2018). Defining optimal length of opioid pain medication prescription after common surgical procedures. JAMA Surg.

[9] Kim N, Matzon JL, Abboudi J (2016). A prospective evaluation of opioid utilization after upper-extremity surgical procedures: identifying consumption patterns and determining prescribing guidelines. J Bone Joint Surg Am.

[10] Brummett CM, Waljee JF, Goesling J (2017). New persistent opioid use after minor and major surgical procedures in US adults. JAMA Surg.

[11] Thiels CA, Ubl DS, Yost KJ (2018). Results of a prospective, multicenter initiative aimed at developing opioid-prescribing guidelines after surgery. Ann Surg.

[12] Tay HP, Wang X, Narayan SW, Penm J, Patanwala AE (2022). Persistent postoperative opioid use after total hip or knee arthroplasty: a systematic review and meta-analysis. Am J Health Syst Pharm.

[13] Johnson SP, Chung KC, Zhong L, Shauver MJ, Engelsbe MJ, Brummett C, Waljee JF (2016). Risk of prolonged opioid use among opioid-naïve patients following common hand surgery procedures. J Hand Surg Am.

[14] Roberts C, Foster D, Shi GG (2019). A collaborative approach to pain control reduces in-hospital opioid use and improves range of motion following total knee arthroplasty. Cureus.

[15] Padilla JA, Gabor JA, Schwarzkopf R, Davidovitch RI (2019). A novel opioid-sparing pain management protocol following total hip arthroplasty: effects on opioid consumption, pain severity, and patient-reported outcomes. J Arthroplasty.

[16] Schneider J, Broome B, Keeley D (2021). Narcotic-free perioperative total knee arthroplasty: does the periarticular injection medication make a difference?. J Knee Surg.

[17] Lombardi AV Jr, Berend KR, Mallory TH, Dodds KL, Adams JB (2004). Soft tissue and intra-articular injection of bupivacaine, epinephrine, and morphine has a beneficial effect after total knee arthroplasty. Clin Orthop Relat Res.

[18] Carlos Rodriguez-Merchan E, Vaquero-Picado A, Ruiz-Perez JS (2019). Opioid-free total knee arthroplasty? Local infiltration analgesia plus multimodal blood-loss prevention make it possible. HSS J.

[19] Kurtz S, Ong K, Lau E, Mowat F, Halpern M (2007). Projections of primary and revision hip and knee arthroplasty in the United States from 2005 to 2030. J Bone Joint Surg Am.

[20] Parvizi J, Miller AG, Gandhi K (2011). Multimodal pain management after total joint arthroplasty. J Bone Joint Surg Am.

[21] Kardash KJ, Sarrazin F, Tessler MJ, Velly AM (2008). Single-dose dexamethasone reduces dynamic pain after total hip arthroplasty. Anesth Analg.

[22] Peters CL, Shirley B, Erickson J (2006). The effect of a new multimodal perioperative anesthetic regimen on postoperative pain, side effects, rehabilitation, and length of hospital stay after total joint arthroplasty. J Arthroplasty.

[23] Post ZD, Restrepo C, Kahl LK, van de Leur T, Purtill JJ, Hozack WJ (2010). A prospective evaluation of 2 different pain management protocols for total hip arthroplasty. J Arthroplasty.

[24] Runner RP, Luu AN, Thielen ZP (2020). Opioid use after discharge following primary unilateral total knee arthroplasty: how much are we over-prescribing?. J Arthroplasty.

[25] Vora MU, Nicholas TA, Kassel CA, Grant SA (2016). Adductor canal block for knee surgical procedures: review article. J Clin Anesth.

[26] Sankineani SR, Reddy AR, Eachempati KK, Jangale A, Gurava Reddy AV (2018). Comparison of adductor canal block and IPACK block (interspace between the popliteal artery and the capsule of the posterior knee) with adductor canal block alone after total knee arthroplasty: a prospective control trial on pain and knee function in immediate postoperative period. Eur J Orthop Surg Traumatol.

[27] Kuang MJ, Ma JX, Fu L, He WW, Zhao J, Ma XL (2017). Is adductor canal block better than femoral nerve block in primary total knee arthroplasty? A GRADE analysis of the evidence through a systematic review and meta-analysis. J Arthroplasty.

[28] Bijur PE, Latimer CT, Gallagher EJ (2003). Validation of a verbally administered numerical rating scale of acute pain for use in the emergency department. Acad Emerg Med.

[29] Foster D, Shi G, Lesser E, Heckman MG, Whalen J, Forte AJ, Wilke BK (2019). A prospective, blinded study comparing in-hospital postoperative pain scores reported by patients to nurses versus physicians. Cureus.

[30] Shah A, Hayes CJ, Martin BC (2017). Factors influencing long-term opioid use among opioid naive patients: an examination of initial prescription characteristics and pain etiologies. J Pain.

[31] Wilke BK, Foster DT, Roberts CA (2019). Early in-hospital pain control is a stronger predictor for patients requiring a refill of narcotic pain medication compared to the amount of narcotics given at discharge. J Arthroplasty.

[32] Chen EY, Betancourt L, Li L, Trucks E, Marcantonio A, Tornetta P 3rd (2020). Standardized, patient-specific, postoperative opioid prescribing after inpatient orthopaedic surgery. J Am Acad Orthop Surg.

[33] Wyles CC, Hevesi M, Trousdale ER (2019). The 2018 Chitranjan S. Ranawat, MD Award: developing and implementing a novel institutional guideline strategy reduced postoperative opioid prescribing after TKA and THA. Clin Orthop Relat Res.

